# Palliative Care for Cancer Patients in Asia: Challenges and Countermeasures

**DOI:** 10.3389/or.2023.11866

**Published:** 2024-01-16

**Authors:** Yu Wang, Xinqing Zhang, Yilin Huang, Xiangyu Ma

**Affiliations:** ^1^ Department of Radiation Oncology, National Cancer Center/National Clinical Research Center for Cancer/Cancer Hospital, Chinese Academy of Medical Sciences and Peking Union Medical College, Beijing, China; ^2^ School of Humanities and Social Sciences, Peking Union Medical College, Beijing, China; ^3^ Division of Prevention and Community Health, National Center for Cardiovascular Disease, National Clinical Research Center of Cardiovascular Disease, State Key Laboratory of Cardiovascular Disease, Fuwai Hospital, Chinese Academy of Medical Sciences and Peking Union Medical College, Beijing, China

**Keywords:** palliative care, hospice care, cancer, personalized medicine, review

## Abstract

With the increasing incidence of cancer worldwide, palliative care has become an effective intervention to relieve cancer patients’ pain and improve their quality of life, although the present development of palliative medicine and hospice care in many Asian countries remains insufficient. To this end, this review comprehensively discussed the main challenges that influence the promotion of palliative medicine, from the perspective of both healthcare professionals and cancer patients. We further proposed and summarized a series of potentially effective countermeasures and solutions, including the shared decision-making modal, multidisciplinary professional cooperation, application of modern science and technology, standardization training for medical workers, personalized palliative treatment regimens, and others, aiming to improve the clinical quality of palliative care practice for cancer patients and promote the development of palliative medicine in Asian regions.

## Introduction

In recent years, with the rapid development of Asian economy and medicine, the spectrum of clinical diseases has undergone significant changes. The incidence of parasitic diseases and other infectious diseases in Asia had greatly reduced, while the incidence of malignant diseases is increasing [1–3]. Cancer is the leading cause of global disease burden [4]. The clinical features of cancer include difficulty to cure, rapid progression and higher medical cost. Thus, cancer diseases could bring a huge physical and mental burden to patients and their families [5].

Palliative care, as an important component of people-oriented health services, aims to alleviate severe suffering related to disease and improve the quality of life, and has become an effective intervention widely used in the treatment of cancer [6]. However, the development of palliative care for cancer patients in many Asian regions remains incomplete and inadequate [7, 8]. The awareness and knowledge of palliative medicine and advanced care planning were at low levels even in the diverse Asian groups living in the United States, which raised concerns and demonstrated the urgent need for appropriate education programs on palliative care in Asia [9]. The purpose of this review is to comprehensively explore the specific factors and challenges influencing cancer palliative care in Asian regions and further propose potential countermeasures with clinical practical significance.

The academic term “palliative care” was first used by a Canadian physician named Balfour Mound in 1974, when he incorporated principles from the British hospice movement into acute care hospitals in Canada first and then around the world [10]. Further three main features were developed in palliative care programs: multidimensional assessment and management of severe physical or emotional distress, professional medical care by multiple disciplines, and emphasis on caring not only for the patients but also for their families [11]. Currently, the academic community generally agrees with the definition revised by the World Health Organization (WHO) in 2016—“Palliative care is a crucial part of integrated, people-centred health services and relieving serious health-related suffering” [12]. Therefore, palliative medicine, also known as palliative care, is not only an effective medical intervention, but a comprehensive method to improve the quality of end-stage life, which has become the consensus of most definitions [13]. At the same time, palliative care also has a holistic view and takes various dimensions into account, including physical, mental, social, spiritual and economical condition of patients, which is especially helpful to those terminally ill patients with advanced cancer, experiencing pain and suffering [14, 15].

The promotion of palliative care is a global ethical responsibility. Patients suffering from a variety of conditions, such as cancer, cardiovascular disease, acute organ failure, severe burns, end-stage chronic disease, severe trauma or extremely fragility, and many other acute or chronic diseases, may all benefit from palliative care to manage their symptoms and improve their quality of life. As such, palliative care should to be emphasized and incorporated into all levels of medical practice over time [16]. According to statistics from WHO, it is estimated that globally only 14% of patients who require palliative care can receive it [12]. In order to attain the Sustainable Development Goals and achieve universal health coverage, countries have to strengthen palliative care services and take it as a key part of health systems.

The definition of “Hospice care” is widely identified by the academic community as follows: Hospice care, including psychological and spiritual supports provided by medical professionals and volunteers, helps the dying person achieve peace, comfort and dignity, and mainly focuses on end-of-life patients, as well as their families [17]. Therefore, hospice care refers to the care and nursing services provided by medical staff, social groups and volunteers for critically ill patients and their families, so that all of them can correctly face and accept the terminal stage of the disease and the death [18].

At present, the application of hospice care, especially in patients with advanced cancer, has received special attention, because it can effectively relieve pain and improve quality of life, while comforting their families and relieving their psychosocial burden caused by cancer. When oncologists proactively discuss end-of-life concerns, patients and their family members tend to choose more comfort-focused care aligned with their values and goals [19]. Furthermore, hospice care—a care service that focuses on end-stage patients and their families—is widely encouraged and gradually integrated into comprehensive cancer treatment regimens nowadays, given the additional integration of cancer services with hospice care will help to provide more seamless care for patients and support for family caregivers during their caregiving and after the death of patients [20].

## Current Status of Palliative Care in Asia

Due to the particularity of cancer, most patients and their families may first encounter the term “palliative care” under the guidance, explanations, and suggestions from their doctors. Currently, the awareness of palliative care is lower in many Asian developing countries, such as China and India, compared to developed countries in Europe and America. Although the Chinese government has increased its support for palliative care in recent years, owing to the lack of education around hospice care and the influence of the traditional Chinese Confucian concept of “filial piety,” many individuals resist palliative medicine, especially in rural areas [21]. In addition, ethnic minority groups in Asia are less likely to discuss issues involving end-of-life treatment preferences and utilize palliative care or hospice services; language barriers, religion, lower levels of health literacy, and limited access to healthcare services and information may contribute to these disparities [22]. A latest cross-sectional study by Yu et al. [23] identified insufficient knowledge and inappropriate behaviours in breakthrough cancer pain diagnosis, assessments, and management among practitioners providing palliative care in Shanghai, China, highlighting an urgent need to improve the quality of cancer palliative care practice in this region.

Similarly, in Thailand, Wongprom et al. [24] demonstrated the gap between expected care and actual care in grief management and spiritual care, though they have been recognized as essential domains in palliative care practice. Grief and bereavement care were mentioned in the residency curriculum in Thailand, whereas there were contradictions and inconsistency with respect to spiritual care practice [24]. Meanwhile, Prachanukool et al. [25] further suggested the early integration of palliative care and hospice consultation into the clinical management for patients at their end-of-life stages, which reflects the conceptual development and practical improvements on palliative care in Thailand.

Hence, despite the underdeveloped *status quo* of palliative medicine in multiple Asian countries, palliative and hospice care has been greatly promoted on the account of the rapid progress of Asian medical and health undertakings [26]. For example, the community-based palliative care model has been recently established in Busan city, Korea, based on principles of palliative medicine and characteristics of public health, aiming to develop the medical support system, increase professional personnel capacity, and improve the quality of palliative care in Korea [27]. Likewise, in Japan, providing palliative care is one of the most important health issues, and palliative medicine in Japan has also progressed rapidly, with two major specialized palliative services: palliative care units and hospital palliative care consultation teams [28]. At present, the number of palliative care units in Japan is 215 (involved in 8.4% of all cancer deaths), and there already are approximately 500 hospital palliative care teams [28]. In India, palliative care providers lobbied to gain access to methadone for pain relief and this has finally been achieved in recent years, which also represented a progress of palliative care, to some extent [29]. Palliative care activists in India counted on numerous strengths, including the various national and state government initiatives that have been introduced recognizing the importance of palliative care. Moreover, National Network of Palliative Care is one of the largest community-driven palliative care programs in the world, with branches in more than ten states in India to date [29, 30].

Although Singapore has established itself as a leading multifaceted medical hub and continues to pursue excellence in palliative care, Loh et al. [31] observed insufficient integration of allied health and rehabilitation services, passive engagement of community partners, lack of specialized skill sets and knowledge in cancer supportive and survivorship care in Singapore. Additionally, a retrospective review of 683 patients died without intensive care unit admission in a Singaporean hospital, which evaluated the prevalence of do-not-resuscitate (DNR) orders, revealed a lack of commitment by doctors on orders for both DNR and to limit life-sustaining therapies [32]. Even in the Asian developed country, Singapore, there were infrequent discussions with patients on end-of-life clinical decisions and excessive burdensome interventions for the dying [32]. Hence, the appropriate and high-quality palliative care practice is still somewhat lacking in Asian developed countries, and should be attached more importance in the future. The *status quo* of palliative care in the different Asian countries is summarized in Table 1.

**TABLE 1:** The current situation of palliative care in several Asian countries.

Country	Current status of palliative care
China	Underdevelopment, in conflict with some traditional values, insufficient palliative care knowledge and skills, but increasing support from government.
Thailand	Generally recognized in theory, but there are contradictions and inconsistency in practice.
Korea	Rapid development in the community-based palliative care and related support systems.
Japan	Widespread attention to palliative care practice and unremitting efforts to promote specialized palliative care services.
India	Active social engagement and projects on palliative medicine.
Singapore	Relatively developed, despite the insufficient integration into oncology treatments and inappropriate palliative care practice.

## Challenges of Palliative Care Practice in Asian Regions

### Conflicts of Traditional Values in Asians

When scholars explored the obstacles in developing palliative medicine practice, they found that the individual conflicts of traditional values might be the main factor in Asian regions [32]. For instance, in ancient Chinese culture, the death or illness was deemed unlucky, and people would be as much as possible to avoid discussions regarding “death”. Besides, the traditional Chinese Confucian concept of “filial piety” may also mislead patients to choose inappropriate radical treatment at an inappropriate time, and thus let them become rejective to palliative medicine [21, 33]. These negative attitudes and avoidance to end-of-life topic could against the education and promotion of palliative care, to a large extent. Therefore, an attitudinal shift regarding the pain and illness is in urgent need through extensive and inspiring public educational activities in India and many other Asian developing countries [29]. In India, studies indicated that patients’ experiences of spirituality are determined by the specifically Indian traditional context, which also gives rise to particular ethical issues and challenges in palliative care [34].

At the same time, the alienation and the war metaphor of disease have deepened the dualism of medical outcome evaluation, probably leading individuals to mistake hospice care for a “failed and abandoned” choice [35]. The misperception of palliative medicine as giving up could leave undesirable impression on patients and their families in some Asian regions, negatively influence the public acceptance, deepen their fear of illness, and result in adverse impacts.

### Limited Availability of Medical Recourse

Recent studies have showed that community health services remain inadequate in China, especially with respect to home-based palliative care, albeit the majority of cancer patients have a preference of home death [36, 37]. As such, limited resources of palliative care would inevitably affect the choice and acceptance of patients and their families. In addition to China, home hospice is inconsistently available in many other Asian countries, and very few of them have enough palliative care specialists to meet their current workforce needs, let alone meet anticipated future needs [38]. For instance, although opioids are accepted as essential for cancer pain treatment globally, the accessibility and availability of this medicine are very limited in India as in various parts of the developing world [29].

Of note, on 15 April 2006, the establishment of China Life Care Association (CLCA) marked the arrival of the spring of hospice care in China. The comprehensive oncology care service of Hong Kong University-Shenzhen Hospital (HKU-SZH), which was established in 2012 as a new model of publicly funded healthcare in mainland China, achieved a satisfactory level of end-of-life care in patients with advanced cancer [39]. Since previous studies indicated inappropriate and inadequate palliative care practice even in Singapore [31, 40], in addition to intensified public education, completing supportive policy, and increasing palliative care facilities in developing countries, public availability to high-quality palliative care services should be emphasized in Asian developed countries when it comes to promoting palliative medicine.

### Clinical Expertise of Physicians

Nowadays the clinical medical staff remain the major practitioner of palliative care worldwide [41]. Thus, ideally district hospitals should train doctors, nurses, pharmacists, and social workers in primary palliative care centres. In addition, a well-defined curriculum and educational program is also needed to ensure uniform training for palliative care providers, when the palliative consultation especially for cancer patients has significantly increased in recent years [42]. When physicians are going to adopt palliative care towards patients with advanced cancer or other end-stage conditions, their professional knowledge, clinical skills and positive attitude are necessary. In daily clinical oncology practice, it is impossible to provide a safe, reliable and high-quality palliative care planning without expertise. In consequences, clinicians need to accumulate the practical experience and professional knowledge of palliative care, constantly popular the public education and promote the general understanding of palliative care in Asian regions. Moreover, drawing on the useful experience from Western developed countries, it is important to emphasize palliative care in clinical practice guidelines, along with regulation, training and certification of care providers, to ensure reliable access to palliative care in Asia.

### Family Support and Socioeconomic Background

In recent years, with the economic development and life quality improvement, great changes have taken place in many Asian countries. For example, the incidences of infectious and parasitic diseases have significantly reduced, and incidences of malignant tumours are growing [1, 2]. Cancer diseases are difficult to cure, causing huge economic burden and social pressure, and aggravating the physical and mental pain of patients. At the same time, palliative and hospice care for cancer patients require not only sufficient medical resources from doctors, but also abundant human resources input from patient families. Studies indicated that family caregivers provided substantial care for patients with advanced cancer, while suffering from hidden morbidity and unmet needs [43]. As a direct consequence of assuming the caregiver role, cancer family caregivers play an important role in the palliative care, but also are at increased risk for physical and mental pressure in hospice and bereavement phases [44, 45]. Therefore, in addition to treatment willingness of cancer patients, the understanding and supports of their families and social networks, as well as their socioeconomic background, both are crucial factors influencing the decisions on palliative care.

## Countermeasures to Promote Palliative Care in Asia

### Shared Decision-Making Model

In order to help cancer patients and their families make appropriate decisions with respect to palliative care, medical staff, patients and their families need to openly discuss the objective condition, true treatment willingness of patients, their medical demands and specific treatment options, so as to further prepare for the doctor-patient cooperation. Meanwhile, the nursing staff, as health educators, palliative caregivers and medical information conveyer, should also be actively involved in the medical decision-making work, aiming to provide high-quality palliative care. Obviously, healthcare professionals are trained to be expert in planning treatment regimens, yet patients are more aware of their lifestyle, environment, financial situation and how much they can rely on home care resources [46]. As a result, the shared decision-making model is becoming more and more important in modern medicine. On the basis of effective communication and cooperation between doctors and patients, the shared decision-making model for palliative care will achieve the optimal clinical results.

More importantly, medical workers should fully respect and understand the choices of patients. The appropriate end-of-life care measures are based on the willing acceptance and approval of patients and their families [47]. Only in this way can medical interventions achieve maximum clinical benefits. Given the substantial development of co-decision in clinical practice, especially in the field of cancer treatment, this beneficial healthcare model also should be applied and promoted in palliative medicine.

### Professional Multidisciplinary Cooperation

Palliative treatment and hospice care for patients with advanced cancer have been concerned by more and more clinicians, humanistic ethics scholars and social activists. In 2014, World Health Assembly (WHA) adopted a landmark resolution calling on all member states to take palliative care as part of comprehensive cancer treatment, and to give it sufficient attention and practical application in cancer multidisciplinary treatment [48, 49].

At present, the multidisciplinary team involvement is commonplace in many treatment settings across the world. Teamwork is also perceived by many experts as an indispensable functionality of palliative care teams [50]. Among the palliative care models carried out in Asian regions, most of them were as follows: 1) Professional palliative care institutions and program: the community ward based on multidisciplinary program, with medical care and entertainment facilities (such as Indian National Program in Palliative Care, The Busan Community-based Palliative Care Project in Korea) [29, 51]; 2) Palliative wards in general hospitals: using hospital resources to provide medical service, nursing and supportive care for terminally ill patients, based on multidisciplinary cooperation (such as the department of palliative and hospice care of West China Fourth Hospital, department of palliative medicine of Tsukuba Medical Center Hospital in Japan, and department of palliative medicine of Tan Tock Seng Hospital in Singapore) [52–54]; 3) Specialized palliative medical units established in cancer hospitals: providing palliative care services for cancer patients (such as the palliative care centre of Peking University Cancer Hospital in China) [55].

To sum up, although the specific presentation model of palliative medicine varies, the successful healthcare services are always on the basis of the multidisciplinary team cooperation. Therefore, it is essential to attach importance to multidisciplinary professional medical team, in order to provide more comprehensive palliative treatment with high quality in Asian regions.

### Clinical Utility of Modern Technology

Despite the high potential to improve the quality of life, palliative care services face significant obstacles to their use in some Asian regions, probably due to limitations of technology and economics in some Asian developing countries [56]. Nowadays, with artificial intelligence (AI), machine learning, robotic surgery and the Internet big data, more and more clinical work can be replaced by the modern high technology. These modern technologies are now applied to various clinical medical practice [57]. In the field of palliative medicine, the combination of modern scientific technologies with the careful care and professional expertise from medical staff may further facilitate the development of palliative care in Asia.

As a consequence, in order to advance palliative medicine and optimize palliative care services utilization in Asian regions, the combination of technology and humanity, as well as the subjective and objective application of medical progress brought by high-technology improvements, both are of great value. Additionally, a shift in public perspective, concerning the use of modern technology in palliative care managements, also should be encouraged. Because based on the modern scientific technology, clinicians are more likely to provide improved high-quality palliative care for cancer patients and their families, and accordingly better help them face the end-of-life diseases and death.

### Personalized Palliative Treatment

Given different physical and psychosocial condition of every cancer patient, the personalized palliative care treatment regimen should be adopted in Asian countries with corresponding medical conditions. To this end, emphasizing accurate diagnoses, appropriate therapeutic regimens and corresponding adjustment according to the real-time situation is of great value in developing palliative care level in Asian regions [58].

Currently, personalized medicine based on tumour characteristics is transforming the management of cancer. Personalized oncology is evidence-based, individualized medicine that delivers the right care to the right cancer patient at the right time and thus results in measurable improvements in outcomes and further reductions on medical costs. In the future, palliative care givers should focus more on every individual patient, using the optimal and personalized treatment regimen to manage pain, other uncomfortable symptoms and negative emotions. More importantly, in the light of different values, beliefs, social and economic background of patients and their families, caregivers should attach importance to meet their subjective needs and guide them to correctly face the cancer disease. To provide patients all-round positive palliative care, medical staff ought to change the key point from “disease-oriented” to “patient-centred.” Only on this basis can we enhance the quality of palliative care for cancer patients, which is conducive to the progress and development of palliative care practice in Asian regions.

### Others

Lastly, there were also some other potential measures and strategies to foster the development of palliative care in Asian regions, such as developing standardized structures for palliative care practice in the community or regional palliative care projects, proposing professional palliative care models suitable for cancer patients, establishing objective methods to measure the quality of palliative care at a national level, and introducing more evidence-based hospice care and palliative medicine to the public and the policymakers [28]. Furthermore, sufficient and appropriate supportive strategies are particularly conducive to patients with advance cancer, such as anticipating, preventing and treating suffering throughout the continuum of illness, and effectively addressing symptom distress. Besides, eliciting value-based goals of cancer care, respecting the DNR wishes of patients and hold to the commitment, allowing withholding or withdrawal of life sustaining measures, and attaching more importance to bereavement support are all vital to cancer patients and their families [31].

## Discussion

Taken together, current development of palliative care in most Asian countries remains insufficient, probably due to some traditional value conflicts, limited availability of palliative care, inadequate training in palliative medicine for medical personnel, lack of family supports and finite socioeconomic background of patients. In order to improve the quality of hospice care for cancer patients and their families in Asia and promote the development of palliative care, countermeasures such as shared decision-making, multidisciplinary cooperation, standardized palliative care training for medical workers, clinical application of modern scientific techniques, personalized palliative treatments, and other potential strategies should be adopted. As such, palliative care practice for cancer patients will be improved in Asia.
